# Development of a disease-specific health utility score for chronic obstructive pulmonary disease from a discrete choice experiment patient preference study

**DOI:** 10.1017/S0266462324000242

**Published:** 2024-05-02

**Authors:** Byron Jones, Mandy Ryan, Nigel S. Cook, Florian S. Gutzwiller

**Affiliations:** 1 Patient Engagement Science, Novartis Pharma AG, Basel, Switzerland; 2 Health Economics Research Unit, University of Aberdeen, Aberdeen, UK; 3 Global Patient Engagement, Novartis Pharma AG, Basel, Switzerland; 4 Global Value & Access, Novartis Pharma AG, Basel, Switzerland

**Keywords:** disease-specific health utility score, chronic obstructive pulmonary disease, EQ-5D-3L, patient preference

## Abstract

**Objectives:**

While patient input to health technology assessment (HTA) has traditionally been of a qualitative nature, there is increasing interest to integrate quantitative evidence from patient preference studies into HTA decision making. Preference data can be used to generate disease-specific health utility data. We generated a health utility score for patients with chronic obstructive pulmonary disease (COPD) and consider its use within HTAs.

**Methods:**

Based on qualitative research, six symptoms were identified as important to COPD patients: shortness of breath, exacerbations, chronic cough, mucus secretion, sleep disturbance, and urinary incontinence. We employed a discrete choice experiment (DCE) and the random parameter logistic regression technique to estimate utility scores for all COPD health states. The relationship between patients’ COPD health utility scores, self-perceived COPD severity, and EQ-5D-3L utility scores was analyzed, with data stratified according to disease severity and comorbidity subgroups.

**Results:**

The COPD health utility score had face validity, with utility scores negatively correlated with patients’ self-perceived COPD severity. The correlation between the COPD health utility scores and EQ-5D-3L values was only moderate. While patient EQ-5D-3L scores were impacted by comorbidities, the COPD health utility score was less impacted by comorbid conditions.

**Conclusions:**

Our COPD utility measure, derived from a DCE, provides a patient-centered health utility score and is more sensitive to the COPD health of the individual and less sensitive to other comorbidities. This disease-specific instrument should be considered alongside generic health-related quality of life instruments when valuing new COPD therapies in submissions to licensing and reimbursement agencies.

## Introduction

There is growing interest in more patient-focused drug development, including a greater recognition during the licensing and reimbursement process of what matters most to the patient and the value to the patient offered by new technologies (1–3). Multi-stakeholder collaborations such as IMI-PREFER have explored how patient preferences can inform decision making across the product lifecycle and generated guidelines to facilitate this (4).

While patient input to health technology assessment (HTA) has traditionally been of a qualitative nature (testimonials, patient submissions, questionnaires, or participation of individual patients during HTA meetings (5;6)), there is a call for more integration of (quantitative) evidence from patient preference studies into HTA decision making (6–9). While some HTA bodies have already reflected on how data from patient preference studies might feed into their processes (10), this is still an open and evolving area of research. Various possibilities have been assessed for how patient-based evidence from preference studies may best be utilized to inform HTA decision making (11;12).

Generic health-related quality of life (HRQOL) instruments like EuroQOL’s EQ-5D (13), are used by various HTA bodies as the primary means of calculating health utility scores. However, the generalizability of EQ-5D, which enables it to be administered to patients with different diseases, may, however, mean that it misses some of the more subtle HRQOL consequences of a disease. Notwithstanding such limitations, the National Institute for Health and Care Excellence (NICE) has stated recently that EQ-5D-3L remains their preferred measure for HRQOL determination in adults (14). Furthermore, Bouvy et al. (10) have stated that, while patient preferences are one of NICE’s priority areas for methods research, they do not currently see a role for the direct integration of patient preference data into economic models. They suggest that further research is needed before these studies can be adopted into NICE’s methods and processes (10).

We describe how the results of a patient preference study might be used to generate disease-specific utility values and inform HTA decision making, alongside more traditional quality of life and cost effectiveness submission materials. Our focus is the development of a disease-specific utility score for chronic obstructive pulmonary disease (COPD), one of the leading causes of morbidity and mortality worldwide (15). COPD is a progressive disease characterized by persistent respiratory symptoms and airflow limitation, causing significant morbidity and mortality; it is associated with economic, societal, and personal burden at all stages (16–20), resulting in high rates of emergency department visits, hospitalizations, and readmissions (21–24).

Our previously reported patient preference study used a discrete choice experiment (DCE) to explore the relative importance COPD patients place on six symptoms of their disease: shortness of breath, exacerbations, cough, excess mucus, sleep disturbance, and urinary incontinence (25). We use these DCE data to develop a COPD health state utility score, assess the convergent validity with self-perceived severity of COPD, and compare this disease-specific utility score with that of the generic HRQOL instrument, EQ-5D-3L (14).

Our results provide compelling evidence of how DCE preference-based utility data might complement and be used alongside conventional HRQOL determination in submissions to HTA bodies. The combined use of disease-specific and generic instruments provides a deeper insight into the value determination for new drugs, enabling an appreciation of how the disease in question contributes to a patients’ overall quality of life and the utility benefit that can be expected from new therapeutic interventions. We believe that this research helps in clarifying the role that patient preference-based utility data can play in contributing to economic assessments by HTA bodies such as NICE (10).

## Methods

### Patient preference study – DCE

The study used an online DCE supplemented with patient-reported questionnaires, including self-perceived severity of COPD and EQ-5D-3L. We enrolled 1050 COPD patients from five countries: Australia, France, Japan, United Kingdom, and United States, with sample sizes of 150, 150, 150, 200, and 400, respectively (see (25) for more details of the DCE design). In summary, attributes and levels (shown in columns 1 and 2 of Table 1) were derived by qualitative patient research (social media listening (26), use of online bulletin boards, (27) and published literature), with input from patient groups, clinical experts, and scientific advice from NICE (10;25). Experimental design methods were used to derive 11 choice sets; each choice presented two hypothetical COPD patients (A and B) (28). For each of the 11 choice sets, respondents were asked to select the patient they would prefer to be. The respondent guidance indicated that this could mean selecting a profile of a patient who is in a worse condition than the one they are currently experiencing. An example of a choice set is shown in Supplementary Table S1.

**Table 1.** Attributes, levels, parameter estimates, and utility weights

Attribute	Level, definition, and utility model label	Parameter (see Equation 1)	RPL estimate	Std error	Std deviation	95% Confidence interval	COPD utility weight
Shortness of breath	1. Shortness of breath experienced during strenuous activity (e.g., walking uphill/upstairs)	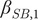	0.881	0.059	1.190	(0.766, 0.997)	0.186
	2. Shortness of breath experienced during light activity (e.g., a short walk on level ground)	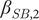	0.717	0.056	0.948	(0.607, 0.828)	0.152
	3. Shortness of breath experience when washing (e.g., taking a shower) or dressing	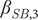	0.425	0.054	0.566	(0.320, 0.530)	0.090
	4. Shortness of breath experienced at rest (e.g., when sitting or lying down)^a^	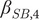	0	–	–	–	0
Cough	1. Cough does not interrupt/disturb any of your usual activities	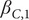	0.408	0.044	0.672	(0.321, 0.496)	0.086
	2. Cough interrupts/disturbs some usual activities.	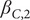	0.198	0.043	0.394	(0.114, 0.282)	0.042
	3. Cough interrupts/disturbs most usual activities^a^	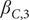	0	–	–	–	0
Incontinence	1. COPD symptoms do not cause any urine leakage	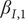	0.787	0.050	1.093	(0.688, 0.885)	0.166
	2. COPD symptoms are causing a few drops of urine leakage	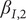	0.562	0.046	0.707	(0.473, 0.651)	0.119
	3. COPD symptoms are causing urine leakage which makes underwear wet^a^	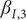	0	–	–	–	0
Mucus clearance	1. It is not at all difficult to bring up mucus	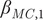	0.614	0.047	0.692	(0.522, 0.706)	0.130
	2. It is a little difficult to bring up mucus	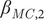	0.436	0.045	0.548	(0.347, 0.525)	0.092
	3. It is very difficult to bring up mucus^a^	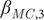	0	–	–	–	0
Sleep disturbance	1. On waking feel rested	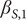	0.914	0.051	1.257	(0.814, 1.013)	0.193
	2. On waking feel somewhat rested	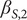	0.693	0.048	0.856	(0.600, 0.786)	0.147
	3. On waking do not feel rested at all^a^	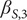	0	–	–	–	0
Exacerbations	1. Never experience any COPD flare–ups/exacerbations	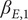	1.129	0.054	1.702	(1.022, 1.235)	0.238
	2. Experience one or more COPD flare–ups/exacerbations that require antibiotics/steroids	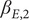	0.731	0.047	1.069	(0.639, 0.823)	0.154
	3. Experience one or more COPD flare–ups/exacerbations that require a hospital stay or visit^a^	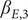	0	0	–	–	0

a Worst level of attribute, and reference level in utility model.

Abbreviations: COPD, chronic obstructive pulmonary disease; RPL, random parameter logistic.

Prior to completing the DCE questionnaire, each patient completed a self-assessment of their current COPD health status using the same attributes and levels as in Table 1. Each patient also rated their own COPD symptoms as either” Mild”, “Moderate”, “Severe”, or “Very Severe” and completed the EQ-5D-3L questionnaire on their current health status. Information was also collected on other comorbidities the patient was experiencing.

### Statistical analyses

Econometric techniques were used to analyze the DCE response data and provide estimates of the utility scores. In particular, the random parameter logistic (RPL) model was used, with the following equation estimated:
(1)

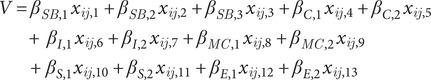

where V is the utility score for any defined health profile, 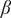 *_i_* (*i* = 1–13) are the parameters of the model to be estimated, and the 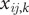 variables define the attribute levels as defined in column 3 of Table 1. All attributes are modeled as dummy variables, with the worse level of each attribute used as the reference, resulting in 13 explanatory variables. The state with levels coded as 433333 is the base comparator, with all attributes at the worse level. Given the inclusion of dummy variables, this model does not impose an interval scale or ordinality on the relationship between the attributes and utility.

The COPD health utility scores for each level of each attribute were calculated using a previously published method (29). For the construction of the COPD health utility score, and to ensure the best level of all attributes resulted in a score of ‘1’, the coefficients were rescaled, while maintaining internal comparisons (ratios). This process followed two stages:

1. The six coefficients that represent the *best* level of each attribute are added to give a total that is labeled *total_best*, that is, *total_best* = 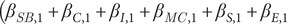 ),
2. All 13 coefficients in Equation (1) are then scaled by dividing them by *total_best*, that is, 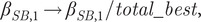 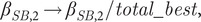 …, 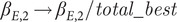 .

The R package *mlogit* (30) was used to fit the RPL model using the data aggregated over the five countries. All model coefficients 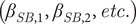 were assumed to be normally distributed. To investigate any differences between countries in terms of preferences, the RPL model was fitted separately to the data from each country.

Patient-level COPD health utility scores were compared to self-perceived health using boxplots, and EQ-5D-3L utility scores (calculated with appropriate country tariffs) were compared to COPD health utility scores using the Spearman correlation coefficient. We also investigated how comorbid conditions impacted on both COPD health utility and EQ-5D-3L scores.

## Results

### Preference parameters

**Figure 1.**
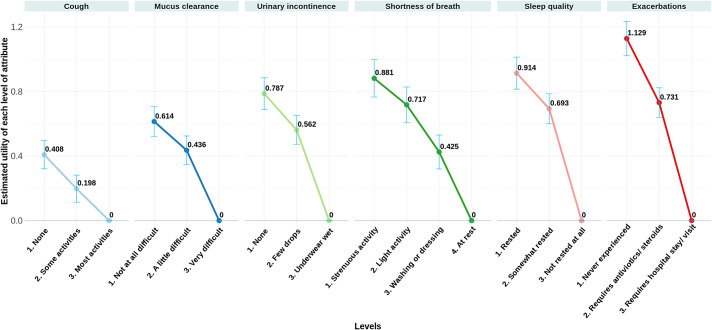
Preference utility estimates obtained from the RPL analysis. Graphical presentation of the preference weights shown as estimates derived from the random parameter logistic (RPL) model. The data are dummy coded with the level with the lowest preference weight for each attribute set to zero. Panels are ordered from left to right by the increasing relative importance of each attribute.

The RPL estimated preference parameters and their standard errors are given in columns 4 and 5 of Table 1, and a graphical display is shown in Figure 1. All estimated preference coefficients are significantly different from zero, indicating that all attributes are important to patients when making a choice. Estimated coefficients have face validity, increasing as the levels move from worst (reference) to better levels. For example, moving from the worst level of shortness of breath (utility zero), utility increases successively from 0.425 to 0.717 to 0.881 (for the best level). Similarly, moving from the worst level of cough (utility zero), utility increases successively from 0.198 to 0.408 (for the best level).

The relative importance of the six attributes, ordered from most to least important, is as follows: exacerbations, sleep quality, shortness of breath, urinary incontinence, mucus clearance, and cough, as displayed in Supplementary Figure S1.

As a sensitivity analysis, we also analyzed the data from each country separately. We found some differences in the pattern of preferences between countries; Supplementary Table S2 shows the relative importance of each attribute within each country. Shortness of Breath is ranked as the most important attribute in Australia and France, exacerbations as the most important in Japan and the United Kingdom, and sleep quality as the most important attribute in the United States. However, in general, there was consistency across countries in relative weighting of the attributes and so for all subsequent analyses (COPD health utility scores, comparisons to EQ-5D-3L and analysis of comorbidities), we have pooled the data across countries to provide a more robust sample size for these determinations.

### Generating the COPD health utility scores

Using the estimated coefficients from Table 1, the *total_best* scores were summed (0.881 + 0.408 + 0.787 + 0.614 + 0.914 + 1.129 = 4.733), then divided into all the estimated coefficients: the resulting RPL utility weight for each attribute level is given in the last column of Table 1. For example, a patient recording a health state of 123113 will have a COPD health utility score of 0.551, that is, (0.186+0.042+ 0 + 0.130 + 0.193 + 0).

### Convergent validity of the COPD health utility scores

**Figure 2.**
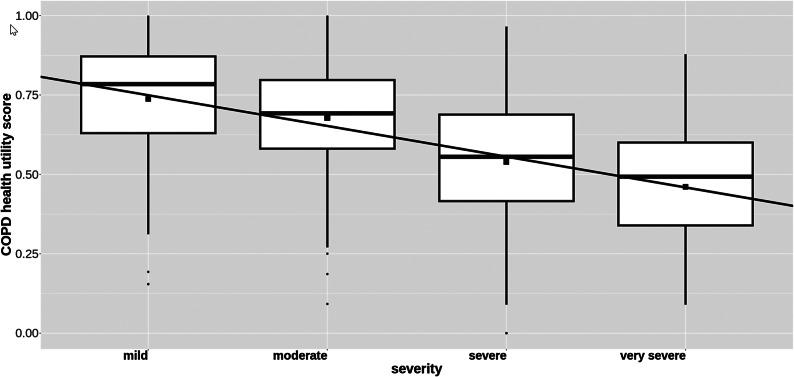
COPD health utility versus self-reported perceived severity of COPD. Boxplots showing the relationship between the COPD health utility score and self-reported perceived severity of COPD. The boxplot for each level of severity shows the median score as a horizontal line and the mean score as a square point. The upper and lower sides of each box indicate the upper and lower limits of the interquartile range of the scores. The vertical lines extending above and below each box have lengths equal to 1.5 times the upper and lower quartile, respectively. Points outside these ranges are plotted individually. The least squares line of best fit has been added to show the declining trend of the means as severity increases. COPD indicates chronic obstructive pulmonary disease.

A boxplot of the relationship between a patient’s COPD health utility scores and self-perceived COPD severity class is shown in Figure 2. The boxplots show that the mean utility score declines as severity increases, indicating face validity. The declining trend in the means has been emphasized by the addition of a fitted least squares line. The least squares line’s negative slope indicates that the COPD health utility scores are negatively associated with the severity categories, with lower COPD utility being associated with greater severity, as expected.

**Figure 3.**
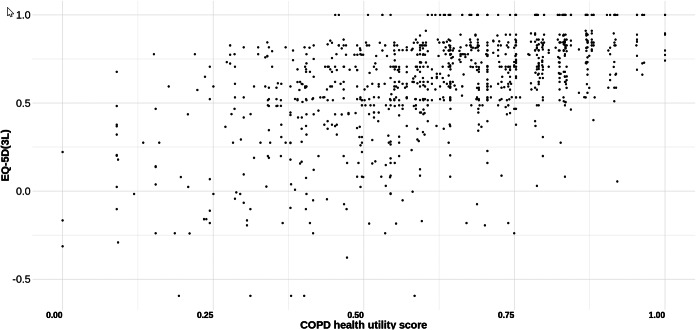
EQ-5D (3L) score versus COPD health utility score for all patients. The coordinates of the points in the figure are the COPD health utility score (*x*-axis) and the ED-5D-3L score (*y*-axis) for each of the 1050 patients in the study. COPD indicates chronic obstructive pulmonary disease.

A plot of the EQ-5D-3L scores (using relevant country tariffs) for all patients and their corresponding COPD health utility scores is shown in Figure 3. The Spearman correlation between the two scores is not strong (0.52 for all patients) and is lower for patients with a positive EQ-5D-3L score (0.48) and much lower for patients with a negative EQ-5D-3L score (0.06).

Supplementary Figure S2 further emphasizes this point: the density plot on the right of the figure shows the spread of values for all patients with a negative EQ-5D (3L) score, with the majority of patients falling in the range from 0 to −0.4 EQ-5D (3L); the density plot on the left, however, shows that for these same patients, the COPD health utility scores are spread fairly evenly across the whole COPD health utility spectrum, with scores from 0 to 0.8.

Given the lack of correlation between the COPD health utility score and EQ-5D-3L for patients with a negative EQ-5D-3L score, we will concentrate on these patients in the rest of the paper.

A closer inspection of the data shown in Figure 3 revealed that there are 12 patients with the worst level on all 5 EQ-5D-3L dimensions (i.e., a code of 33333) and consequently these have the lowest negative EQ-5D-3L scores. However, these 12 patients do not have the worst COPD health utility score of zero but values that range from 0.09 to 0.70, with most values greater than 0.3. This suggests that these patients are not suffering as severely from their COPD, as one might have assumed from their EQ-5D-3L scores.

Looking at the 50 patients that recorded a negative EQ-5D-3L utility score (i.e., not necessarily a 33333 code, but where their score was less than zero when the country tariff was applied), we investigated the comorbidities reported by these patients compared to the patients with the best EQ-5D-3L score (coded as 11111). The results are presented in Table 2, which shows the percentage of patients with each comorbidity out of the total number in each respective EQ-5D-3L subgroup (EQ-5D-3L code =11111, score < =0, or code = 33333). The results clearly show that across all the patient comorbid conditions (anemia, ministroke, congestive heart failure, gastroesophageal reflux disease, malnutrition, osteoporosis, peptic ulcer, vascular, and rheumatological disease) the frequency of the comorbidity increases consistently with worsening EQ-5D-3L scores.

**Table 2.** Comorbidities of patients in EQ-5D-3L subgroups

Condition	EQ-5D-3L code = (11111)	EQ-5D-3L score < =0	EQ-5D-3L code = (33333)
	(%)	(%)	(%)
Anemia	2	30	50
Ministroke	4	16	25
Congestive heart failure	1	20	75
Gastroesophageal reflux disease	10	40	58
Malnutrition	0	12	50
Osteoporosis	5	36	58
Peptic ulcer	1	16	50
Vascular disease	0	20	58
Rheumatological disease	4	36	42
Mean EQ–5D–3L	0.994	−0.183	−0.323
Mean COPD health utility score	0.801	0.378	0.366
Sample size	114	50	12

Abbreviation: COPD, chronic obstructive pulmonary disease.

We further looked at the prevalence of comorbidities within each of the self-perceived COPD severity classes. Supplementary Table S3 shows the percentage of occurrence of each comorbid condition, mean EQ-5D-3L score, and mean COPD utility score within each severity class. The EQ-5D-3L and COPD score means decrease as the COPD severity level increases, as would be expected. However, the prevalence of comorbidities was not found to show a trend toward greater presence of comorbidities with worsening self-perceived COPD severity, indicating that when asked about their COPD disease severity, COPD patients focus on their COPD symptoms and are not being influenced by their other comorbidities.

To better understand the correlation between utility scores and comorbidities, we compared the mean EQ-5D-3L and COPD health utility scores for patients with and without each comorbidity (results shown in Supplementary Table S4). Despite the small sample sizes for some of the comorbidity subgroups, we found that the mean EQ-5D-3L score was always lower than the mean COPD health utility score in patients who presented with a comorbidity. However, in the subgroups that did not have the comorbidity, the means of the EQ-5D-3L and COPD health utility score were found to be very similar. This provides further evidence that the EQ-5D-3L scores are more heavily influenced by comorbidities than the COPD health utility scores.

## Discussion

We have shown in the present study with COPD patients, that a DCE is a sensitive instrument for generating disease-specific health state utility values. The relationship between the COPD health utility estimates and the EQ-5D-3L values determined from the same COPD patients was only moderate, indicating that each has a particular and different message to impart. Most healthcare DCEs are designed as a choice between two or more hypothetical product profiles, with the attributes covering a range of efficacy, safety, and convenience factors for the product profiles in question. Our study differed in that the attributes were based on the disease symptoms that matter most to COPD patients (25–27), allowing the patient preferences for different disease health states to be investigated. Symptom-based preference studies of this kind can be important when conducted early in the medical product lifecycle to define the important clinical endpoints for inclusion in pivotal clinical trials (3;31). Scientific advice was sought from NICE during the design phase of our COPD patient preference study processes (10;25), the outputs of which both led to improvements in the study design and enabled an alignment of stakeholder perspectives around the endpoints that matter most and whose alleviation would constitute the greatest value to the patient (26;27).

The design of the DCE was chosen to maximize the efficiency of estimation of the model coefficients, ensuring that the estimates of the model coefficients would be relatively precise, as can be seen in Table 1. Indeed, all the estimated coefficients were statistically significant from zero, indicating that all attributes were considered important when patients determined their preferences in the DCE. As a robustness check, we compared the RPL results with utility scores estimated from the MNL model. Results, presented in Supplementary Table S5, show the models gave similar results, indicating robustness in the reported utility scores. The robustness of the data from the DCE and the derivation of COPD health utility scores for each patient was further supported by the sensitivity analysis of utility scores according to patients’ perceived severity of their COPD. We also found that comorbid conditions did not seem to impact greatly upon a patient’s estimation of their perceived COPD severity, suggesting that this self-reported severity was indeed specific to their COPD status, not more general health issues.

The literature investigating how to optimally integrate utility scores derived from a disease-specific DCE alongside EQ-5D-3L values is quite limited and remains an important area of scientific investigation (32–36). Burr et al. (29) investigated patient preferences for supporting the estimation of QALY gains as part of a cost-utility analysis. They concluded that their utility scores for glaucoma could be used to populate an economic model for use in a cost-effectiveness analysis. For now, using the COPD health utility score as an informal addition to the EQ-5D-3L score is likely to be an informative step forward in determining the overall and COPD-specific quality of life that is experienced by COPD patients. Indeed, using the COPD health utility score as a complement to that determined through more generic instruments like EQ-5D-3L, will allow a more holistic determination of patient-derived value from new drug treatments.

A key finding from our study is that the correlation observed between the COPD health state utility derived from the DCE and the EQ-5D-3L scores generated from those same COPD patients was only moderate. As reported (25), the average age of the COPD patients in our study was 60.5 years, and it is not surprising to find that they suffered from a range of comorbid conditions, in addition to their COPD. These comorbid conditions did not seem to influence the patients’ self-reported perception of their COPD disease severity, and our analyses would indicate that the COPD health utility scores derived from the DCE were also not influenced to a great extent through the presence of comorbidities. However, the EQ-5D-3L scores derived from these patients were very much influenced by the presence of comorbid conditions (Supplementary Tables S3 and S4). This finding suggests that EQ-5D is influenced to a large extent by comorbidities, as has also been discussed (37), whereas the COPD health utility scores derived from the DCE are more specific to their COPD health status.

This finding has important implications for the generation of utility data in support of HTAs and economic evaluations. Patients in COPD clinical trials are likely to be suffering from a range of comorbid conditions, and hence gathering EQ-5D-3L data from these patients will likely suffer from the same issues as in our study. Moreover, EQ-5D-3L may have limited sensitivity for demonstrating clinical benefits of new investigational drugs for COPD, due to the confounding effect of other comorbidities diminishing their EQ-5D-3L scores. As has also been recommended by others (38;39), we therefore advocate the use of a disease-specific instrument, such as our COPD health utility score derived from the DCE, to be administered alongside the use of EQ-5D-3L in clinical trials; this would be expected to have greater sensitivity for showing the therapeutic benefit of COPD drugs on the symptoms that matter most to COPD patients, without the confounding problem of comorbidities impacting their general health status. The present patient preference study was conducted during the early development of a new therapy for COPD to inform the choice of patient-relevant endpoints to be included in the phase III clinical trial. Unfortunately, the drug in question did not progress beyond phase II. Thus, although an HTA body was involved in providing input to the design of the preference study (10), we will not be able to directly explore the usefulness of this new COPD health state utility score as an input to subsequent HTA submissions.

It seems reasonable to expect that also in other diseases where a generic instrument like EQ-5D fails to fully capture the quality-of-life impact of the disease (38–41), or where comorbid disorders confound the measurement of quality of life (37), then an approach to generating disease-specific utility estimates could be important for determining value to the patient of new technology offerings. The same argument applies to determining those utility estimates with patients suffering from the disease, rather than from the general public, if the quality-of-life impact is not well appreciated by the general population (38;42). This could be of particular importance in the case of rare diseases, where patient preference research is a new and evolving science (42).

Our study has several limitations common to online patient preference elicitation surveys, such as a requirement to access the internet and the patient self-reported completion of the screener to gain access to the survey (rather than via physician referral). Literature would indicate, however, that, even in elderly patients, results from online surveys are consistent with those from other survey administration routes (43;44). Our patients were recruited from patient support groups or COPD patient research panels; while this increases the likelihood that patients were indeed confirmed COPD patients, it does increase the risk that those recruited may have been more engaged with their disease and having a greater interest in their health and management, than the broader COPD population. Caution is therefore needed in extrapolating the results from this study to a broader COPD population. Specific to the analysis in this manuscript of utility scores derived from patient health state preferences, a limitation that is often the case for DCEs is that the fitted model did not contain any terms to account for potential interactions between the attributes. We believe this is not unreasonable given that statistical significance tests for such interactions typically have low power for practical sample sizes.

## Conclusions

Preference studies are increasingly performed to supplement regulatory and HTA submissions (4;11). Our study provides information for decision makers on an approach whereby utilities can be generated from a DCE that are specific to the disease and based on what matters most to those patients. We have shown that a more generic HRQOL instrument is limited in its sensitivity due to the impact of comorbidities suffered by the patients, whereas the disease-specific health state utility scores derived from the DCE are less susceptible to comorbid conditions.

An approach of this kind to deriving disease-specific utilities can inform value and reimbursement discussions on new therapeutic modalities and the extent to which improvements in aspects of their disease, COPD in this example, would translate into patient-derived value from those medications. We hope our study will inspire further research aimed at using patient preference data to derive utility values and support HTA discussions. HTA bodies have called for further research to explore under what circumstances patient preference studies would offer the most added value to HTA (10). We believe that our research sheds light on how disease-specific utility values derived from patient preference studies can complement generic HRQOL instruments in informing HTA discussions on patient value and cost-effectiveness.

## Supporting information

Jones et al. supplementary material Jones et al. supplementary material

## Supplementary material

The supplementary material for this article can be found at http://doi.org/10.1017/S0266462324000242.
