# Supplementary material for: Development of a disease-specific health utility score for chronic obstructive pulmonary disease from a discrete choice experiment patient preference study
Source: Int J Technol Assess Health Care. 2024 May 2;40(1):e30. doi: 10.1017/S0266462324000242 (PMC11569910; doi:10.1017/S0266462324000242)

**Supplementary material for:**

Jones, B., Ryan, M., Cook, N.S. and Gutzwiller, F.S. (2023)

Development of a Disease-Specific Health Utility Score for Chronic Obstructive Pulmonary Disease from a Discrete Choice Experiment Patient Preference Study.

Submitted to *International Journal of Technology Assessment in Health Care*.

**Supplementary Table S1: Example of a DCE choice set**

| **Attribute** | **Patient A** |  | **Patient B** |
| --- | --- | --- | --- |
| **Shortness of breath.**  On a typical day: | You experience **shortness of breath** when **washing (e.g., taking a shower) or dressing** |  | You experience **shortness of breath** when **sitting or lying down** |
| **Cough.**  On a typical day: | Your **cough does not interrupt/disturb any** of your usual activities |  | Your **cough interrupts/disturbs most** of your usual activities |
| **Incontinence.**  On a typical day: | Your COPD symptoms are causing **a few drops of urine leakage** |  | Your COPD symptoms are causing **a few drops of urine leakage** |
| **Mucus.**  On a typical morning: | It is **not at all difficult** to **bring up mucus** |  | It is **a little difficult** to **bring up mucus** |
| **Sleep quality.**  When waking up on a typical morning: | You feel **rested** |  | You **do** **not feel rested at all** |
| **Exacerbations.**  During a typical year: | You **never** experience any COPD flare-ups/ exacerbations |  | You experience **one or more** COPD flare-ups/exacerbations that **require a hospital stay or visit** |

COPD indicates Chronic Obstructive Pulmonary Disease; DCE, Discrete Choice Experiment

**Supplementary Table S2. Relative importance (%) of each attribute in each country**

|  | **Country** | | | | |
| --- | --- | --- | --- | --- | --- |
| **Attribute** | **Australia** | **France** | **Japan** | **UK** | **USA** |
| **Shortness of breath** | 20.44 | 25.07 | 15.82 | 18.69 | 16.42 |
| **Cough** | 12.33 | 11.21 | 5.46 | 4.79 | 9.22 |
| **Urinary incontinence** | 14.88 | 17.80 | 17.58 | 22.40 | 13.68 |
| **Mucus clearance** | 13.43 | 10.69 | 11.34 | 12.69 | 12.71 |
| **Sleep quality** | 18.84 | 16.32 | 24.42 | 14.07 | 24.25 |
| **Exacerbations** | 20.08 | 18.91 | 25.38 | 27.37 | 23.71 |

**Supplementary Table S3: Comorbidities of patients in COPD self-perceived severity subgroups**

| **Condition** | **Mild** | **Moderate** | **Severe** | **Very Severe** |
| --- | --- | --- | --- | --- |
| Anemia (%) | 9 | 5 | 7 | 5 |
| Mini-stroke (%) | 5 | 4 | 4 | 3 |
| Congestive Heart Failure (%) | 10 | 6 | 7 | 12 |
| Gastroesophageal reflux disease (%) | 29 | 19 | 23 | 22 |
| Malnutrition (%) | 4 | 1 | 0 | 5 |
| Osteoporosis (%) | 14 | 13 | 20 | 19 |
| Peptic Ulcer (%) | 5 | 1 | 1 | 0 |
| Vascular disease (%) | 8 | 3 | 3 | 4 |
| Rheumatological disease (%) | 16 | 14 | 17 | 12 |
| Mean EQ-5D-3L score | 0.69 | 0.68 | 0.56 | 0.38 |
| Mean COPD utility score | 0.74 | 0.68 | 0.54 | 0.46 |
| Sample size | 116 | 563 | 297 | 74 |

**Supplementary Table S4: Mean EQ-5D-3L score and mean COPD health utility score within each comorbidity subgroup**

| **Comorbidity** | **Present** | **Sample size** | **Mean EQ-5D-3L** | **Mean COPD utility** |
| --- | --- | --- | --- | --- |
| Anemia | Yes | 66 | 0.42 | 0.55 |
|  | No | 984 | 0.64 | 0.64 |
| Malnutrition | Yes | 15 | 0.10 | 0.46 |
|  | No | 1035 | 0.63 | 0.63 |
| Congestive Heart Failure | Yes | 75 | 0.41 | 0.53 |
|  | No | 975 | 0.64 | 0.64 |
| Gastroesophageal reflux disease | Yes | 823 | 0.56 | 0.59 |
|  | No | 227 | 0.65 | 0.64 |
| Mini stroke | Yes | 45 | 0.44 | 0.59 |
|  | No | 1005 | 0.64 | 0.63 |
| Osteoporosis | Yes | 164 | 0.52 | 0.58 |
|  | No | 886 | 0.65 | 0.64 |
| Peptic Ulcer | Yes | 15 | 0.15 | 0.46 |
|  | No | 1035 | 0.63 | 0.63 |
| Vascular disease | Yes | 41 | 0.33 | 0.53 |
|  | No | 1009 | 0.64 | 0.63 |
| Rheumatological disease | Yes | 157 | 0.49 | 0.58 |
|  | No | 893 | 0.65 | 0.64 |

COPD indicates Chronic Obstructive Pulmonary Disease

**Supplementary Table S5: Multinomial logistic regression (MNL) solution**

| **Attribute** | **Level and level definition** | **MNL Estimate** | **Std Error** | **95% confidence interval** | **COPD utility weight** |
| --- | --- | --- | --- | --- | --- |
| **Shortness of breath** | 1. Shortness of breath experienced during strenuous activity (e.g., walking uphill / upstairs). | 0.510 | 0.035 | (0.442, 0.578) | 0.192 |
|  | 2. Shortness of breath experienced during light activity (e.g., a short walk on level ground). | 0.432 | 0.035 | (0.363, 0.500) | 0.163 |
|  | 3. Shortness of breath experience when washing (e.g., taking a shower) or dressing. | 0.255 | 0.035 | (0.187, 0.323) | 0.096 |
|  | 4*. Shortness of breath experienced at rest (e.g., when sitting or lying down). | 0 | - | - | 0 |
| **Cough** | 1. Cough does not interrupt/disturb any of your usual activities. | 0.216 | 0.028 | (0.161, 0.272) | 0.082 |
|  | 2. Cough interrupts/disturbs some usual activities. | 0.119 | 0.028 | (0.063, 0.174) | 0.045 |
|  | 3*. Cough interrupts/disturbs most usual activities. | 0 | - | - | 0 |
| **Incontinence** | 1. COPD symptoms do not cause any urine leakage. | 0.424 | 0.029 | (0.368, 0.480) | 0.160 |
|  | 2. COPD symptoms are causing a few drops of urine leakage. | 0.328 | 0.028 | (0.272, 0.383) | 0.123 |
|  | 3*. COPD symptoms are causing urine leakage which makes underwear wet. | 0 | - | - | 0 |
| **Mucus**  **clearance** | 1. It is not at all difficult to bring up mucus. | 0.354 | 0.028 | (0.298, 0.410) | 0.133 |
|  | 2. It is a little difficult to bring up mucus. | 0.262 | 0.028 | (0.206, 0.318) | 0.099 |
|  | 3*. It is very difficult to bring up mucus. | 0 | - | - | 0 |
| **Sleep disturbance** | 1. On waking feel rested. | 0.532 | 0.028 | (0.477, 0.588) | 0.201 |
|  | 2. On waking feel somewhat rested. | 0.389 | 0.028 | (0.333 0.444) | 0.146 |
|  | 3*. On waking do not feel rested at all. | 0 | - | - | 0 |
| **Exacerbations** | 1. Never experience any COPD flare-ups/exacerbations. | 0.617 | 0.029 | (0.560, 0.673) | 0.232 |
|  | 2. Experience one or more COPD flare-ups/exacerbations that require antibiotics/steroids. | 0.435 | 0.028 | (0.379, 0.490) | 0.164 |
|  | 3*. Experience one or more COPD flare-ups/exacerbations that require a hospital stay or visit. | 0 | 0 | - | 0 |

**Supplementary Figure S1: Relative importance of attributes based on RPL model**


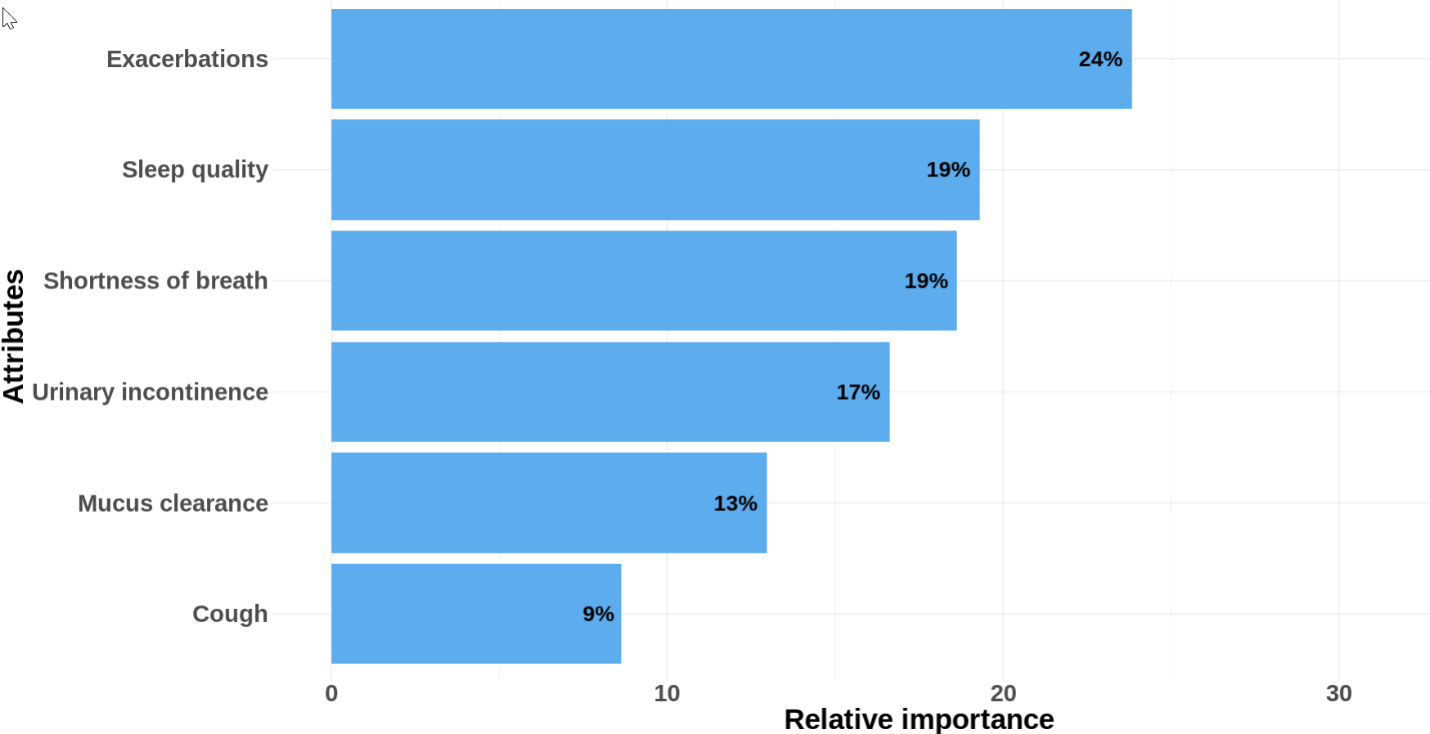


**Supplementary Figure S2:** **Density plots for EQ-5D (3L) and COPD health utility scores for patient with negative EQ-5D (3L) scores**


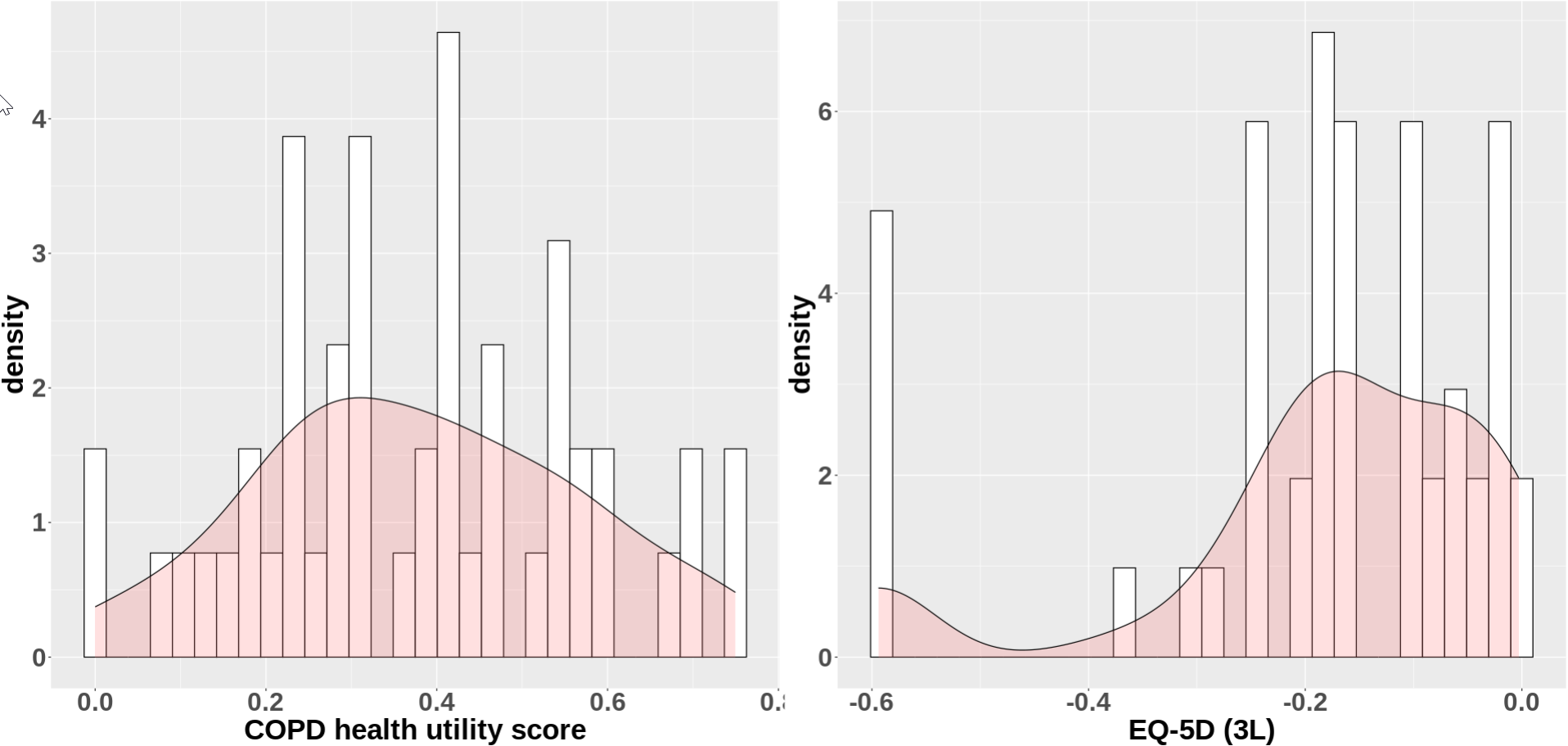

Supplement: Jones et al. supplementary material [file S0266462324000242sup001.docx]
